# Tuning optical and dielectric properties of strontium-zinc ferrite nanomaterials through structure-cation engineering

**DOI:** 10.1038/s41598-025-29798-2

**Published:** 2025-11-25

**Authors:** Osama Tariq Satti, Uzma Ghazanfar, Hassan Wahab, Guoqiang Li, Ahsen Tariq Satti, Aqsa Sohail, Sadiq H. Khoreem, Muhammad Numan Nawaz, Saeed Rehman

**Affiliations:** 1 School of Manufacturing Science and Engineering, Key Laboratory of Testing Technology for Manufacturing Process, Ministry of Education, Southwest University of Science and Technology, Mianyang, 621010 China; 2 Department of Physics, University of Wah, Wah Cantt, 47040 Pakistan; 3 Physics Division, Pakistan Institute of Nuclear Science and Technology (PINSTECH), Islamabad, Pakistan; 4 Department of Physics, Riphah International University, Islamabad, Pakistan; 5 Department of Optometry and Vision Sciences, Al-Razi University, 11623 Sana’a, Yemen; 6 Center of Studies and Research, Yemen, Amran University, Amran, 96711 Yemen; 7 Education Ministry Key Laboratory of Solid Waste Treatment and Resource Recycle, Southwest University of Science and Technology, Mianyang, 621010 China

**Keywords:** Strontium, Nanomaterials, Zinc ferrites, Mössbauer spectroscopy, Material synthesis, Dielectric spectroscopy, Materials science, Nanoscience and technology, Physics

## Abstract

Herein, Sr_x_Zn_1−x_Fe_2_O₄ nanoparticles produced by chemical co-precipitation are examined to determine how strontium (Sr) doping affects their structural, morphological, optical, and dielectric characteristics. The creation of a single-phase cubic spinel structure was verified by X-ray diffraction, and peak shifts indicated lattice expansion brought on by the addition of Sr. A homogeneous grain morphology with nanoscale dimensions was found by FESEM investigation. The metal-oxygen vibrational modes in the Raman and FTIR spectra showed changes that suggested local structural distortion. With a lowered optical bandgap of 2.62 ± 0.1 eV, UV-Vis spectroscopy revealed a red shift in the absorption edge, improving semiconducting properties. AC conductivity and complex modulus tests revealed improved charge transport and relaxation behavior, whereas impedance spectroscopy indicated decreased dielectric loss (tan δ) at high frequencies. Mössbauer spectroscopy revealed that doping resulted in a redistribution of Fe^2+^ between tetrahedral and octahedral sites. According to these results, Sr-doped ZnFeO₄ is a promising material for use in high-frequency and optoelectronic device applications. This work develops a structure-cation engineering approach, where Mössbauer spectroscopy validates cation redistribution, allowing for simultaneous tuning of optical and dielectric properties, providing a new avenue for the development of ferrite-based optoelectronic and energy storage materials.

## Introduction

 In recent eras, metallic oxides have earned the attention of researchers due to their diverse and purposeful applications^1^. Ferrites are typically represented as A_x_B_1−x_Fe_2_O_4_, where A and B are alkaline Earth and transition metals, respectively^2^. Devices fabricated with these materials can absorb microwaves and have military applications for defense systems^3,4^. Due to the spinel and hexagonal ferrite structures, these materials can effectively harness specific ranges of electromagnetic waves in the megahertz to gigahertz range, making it challenging to select a single material as a perfect absorber^5,6^. Zinc oxide (ZnO) exhibits a band gap of approximately 3.2 eV. It is recognized for its stability against chemicals, abundance, and non-toxicity, making it a significant material for semiconducting and photocatalyst applications. In addition, due to the high mobility (200–300 cm^2^V^− 1^s^− 1^) and lifetime (>10 s) of ZnO, an electron can easily migrate from the valence band towards the conduction band and vice versa for the creation of electron-hole pairs^7^. Therefore, these materials have a high signal-to-noise ratio for good fidelity and have low losses at higher frequencies, and hard and soft ferrites, having striking properties, can be solitary solutions^8^.

Strontium Zinc ferrite (SZF), structurally analogous to magnetoplumbite, demonstrates a pronounced magneto-crystalline anisotropy and requires keen attention to make it highly suitable for advanced technological applications^9,10^. They are also used as permanent magnets because of their higher coercivity in recording media for read-and-write heads, telecommunications, microwave, high-frequency, and magneto-optical devices^11^. SZF is ferrimagnetic, in which magnetic moments of disparity sublattices are opposed like antiferromagnetic materials, their opposite moments are unequal, and the atomic radius of Sr, Zn, and Fe is 0.125 nm, 0.138 nm, and 0.126 nm, respectively^12^. In this way, they have a net magnetic moment, through a curie temperature (T_c_) of 732 K, and a distinctive magnetic moment in the range of 17–21 µB per formula unit^13–15^ Cations of iron Fe^3+^ are in an oxidation state, with five diverse surroundings corresponding to the 12k, 4f2, 4f1, 2b, and 2a positions, in the Wickhoff Notation, having different atomic radii, i.e., Fe^2+^ is 0.126 nm, Fe^3+^ is 0.064 nm^16^.

Since many data distributions for ferrites were abnormal, the effects of substituting Fe³⁺ with various transition metals (including Co²⁺, Mn²⁺, Zn²⁺, Ti²⁺, Ni²⁺, and Ir⁴⁺) were thoroughly studied using multiple analytical techniques^17^. When Sunirmal Saha and colleagues investigated terbium-doped nickel ferrite nanoparticles produced using a green sol-gel auto-combustion process, they discovered that these nanoparticles exhibited soft magnetic behavior, decreased dielectric loss, and increased resistivity, making them suitable for high-frequency applications. Properties related to structure, magnetism, and dielectric behavior were also investigated^18^. In related work, Ag-doped NiFe_2_O₄ ferrites produced using a green sol-gel technique showed enhanced conductivity, ferromagnetism, and a smaller band gap, indicating their potential for use in electronic applications^19^. Systematic disparities in resistivity and hyperfine fields drive the stable cubic spinel structure of Ho³^+^-doped Ni-Zn-Co ferrites, which also exhibit reduced dielectric losses and controllable magnetic softness^20^. Mössbauer and spectroscopic investigations confirm that the microwave hydrothermal synthesis of CoZnFe_2_O₄ ferrites produces a single-phase cubic spinel structure with tunable magnetic susceptibility, reduced optical bandgap, and increased cation site preferences^21^.

The structural, optical, and dielectric properties of spinel ferrites substituted with alkaline earth metals like strontium have garnered significant interest. These include Sr_x_Zn_1−x_Fe_2_O_4_ complexes, which offer a promising class of semiconducting oxides with customizable functions for dielectric and next-generation optoelectronic applications. That being said, it is yet unclear how only Sr doping affects the lattice structure, electrical transitions, local bonding environment, and relaxation mechanisms. Crystal symmetry, optical band structure, and dielectric dispersion are all changed by the addition of Sr^2+^ ions, which also cause lattice strain and affect the Fe-O-Fe super-exchange interactions. There is still a lack of a cohesive framework that connects the effects of Sr substitution across crystallographic phase evolution (XRD), vibrational dynamics (FTIR and Raman Spectroscopy), optical absorption (UV-visible), complex impedance (Z′/Z″), dielectric relaxation (ε′/ε″), energy dissipation (tan δ), electric modulus (M′/M″), and AC conduction mechanisms, despite numerous studies on individual conditions^22–48^.

In the present study, we employ a cost-effective and easily accessible chemical precipitation method to synthesize strontium-doped zinc ferrite nanoparticles. This study aims to investigate the influence of Sr in the Zn-Fe environment on its structural, optical, and morphological properties. The distinctive aspect of this work lies in observing iron’s behavior in the presence of cations at various interstitial sites and confirming its semiconducting properties. Sr ions are used as dopants as they have a greater ionic radius, which causes structural deformations and lattice expansion in the spinel framework but also strengthens the super-exchange interaction between Fe^2+^ ions, which improves electrical and dielectric properties, and makes it appropriate for functional applications. Crucially, Sr is safe for the environment, which enables us to achieve our objective of using sustainable, non-toxic materials for said applications. Synthesize Sr-doped Zn ferrite nanoparticles (x = 0 - 0.35) using a simple, scalable chemical precipitation method. In this work, we examine how Sr substitution affects phase evolution, crystallinity, and lattice structure using FTIR and XRD. Using Mössbauer spectroscopy, investigate the oxidation states, magnetic hyperfine interactions, and local Fe cation state. Determine the optical bandgap, electronic transitions, and how these relate to the lattice strain caused by Sr using UV-Vis spectroscopy. Analyze the frequency-dependent dielectric permittivity, electric modulus, and AC conductivity, and relate them to tiny polaron hopping and Maxwell-Wagner relaxation. By synthesizing nanostructured Sr_x_Zn₁₋ₓFe₂O₄ (x = 0–0.35.35) and carefully examining their multi-scale physicochemical properties, this study fills this gap. Frequency-dependent polarization events, which are indicative of Maxwell-Wagner interfacial relaxation and tiny polaron hopping, and structure-induced electronic localization, are found to interact coherently. Low-loss, and environmentally beign ferrites have been the subject of increased research in recent years due to the growing demand for highly efficient, miniaturized, and energy-conserving electronic components. These demands are directly met by SZF, which is a great contender for next-generation semiconductor and dielectric technologies because of its adjustable bandgap, improved polarization, and sustainable manufacturing method. The logical design of ferrite materials with enhanced functions for high-frequency electronics, electromagnetic, and semiconducting platforms is made possible by a thorough understanding.

## Methods and materials

In this study, Strontium Zinc Ferrite (Sr_x_Zn_1−x_Fe_2_O_4_) with x = 0, 0.05, 0.15, 0.25, and 0.35 was fabricated by using the iron nitrate nonahydrate (Fe (NO_3_)_3_.9H_2_O), zinc nitrate hexahydrate (Zn (NO_3_)_2_.6H_2_O), strontium nitrate (Sr (NO_3_)_2_), and sodium hydroxide (NaOH) of Sigma Aldrich having molecular weights 404 g/mol, 189.40 g/mol, 211.63 g/mol, and 40 g/mol respectively. All the chemicals were used directly without further purification. The chemical equation used for this experiment is as follows;

1 $$\begin{array}{l} _{\left( {{\rm{1}} - {\rm{x}}} \right)}{\rm{Zn}}{\left( {{\rm{N}}{{\rm{O}}_{\rm{3}}}} \right)_{\rm{2}}} \cdot {\rm{6}}{{\rm{H}}_{\rm{2}}}{\rm{O }}{ + _{\rm{x}}}{\rm{Sr}}{\left( {{\rm{N}}{{\rm{O}}_{\rm{3}}}} \right)_{\rm{2}}} + {\rm{ 2Fe}}{\left( {{\rm{N}}{{\rm{O}}_{\rm{3}}}} \right)_{\rm{3}}} \cdot {\rm{9}}{{\rm{H}}_{\rm{2}}}{\rm{O }} + {\rm{ 8NaOH }} \to {\rm{ S}}{{\rm{r}}_{\rm{x}}}{\rm{Z}}{{\rm{n}}_{{\rm{1}} - {\rm{x}}}}{\rm{F}}{{\rm{e}}_{\rm{2}}}{{\rm{O}}_{\rm{4}}}\\ {\rm{ }} + {\rm{ 8NaN}}{{\rm{O}}_{\rm{3}}} + {\rm{ gaseous}}~{\rm{by}} - {\rm{products}}~\left( {{\rm{e}}.{\rm{g}}.,~{{\rm{H}}_{\rm{2}}}{\rm{O}},~{\rm{N}}{{\rm{O}}_{\rm{x}}}} \right) \end{array}$$

**Fig. 1:**
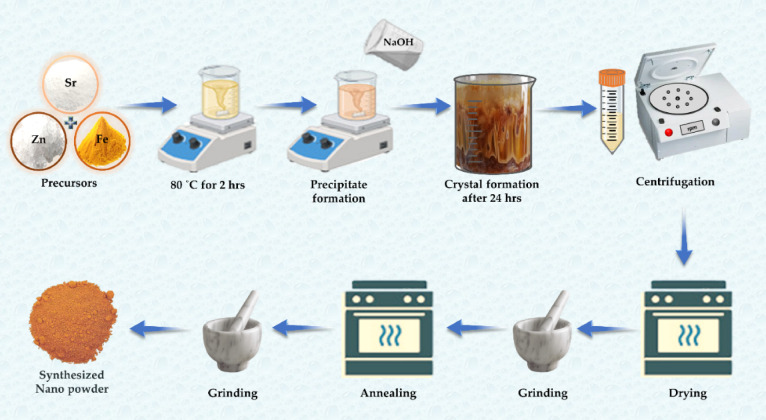
Steps for preparing Sr_x_Zn_1−x_Fe_2_O_4_ by the co-precipitation method.

Primarily, precursors were heated to make an aqueous solution at 80˚C for 2 h. Then, the sample is left at ambient temperature for settlement. After 24 h of settlement, the pH level of the material is maintained at 7 to make it environment-friendly by washing it with deionized water using a centrifuge machine. Next, the specimen is placed in a drying furnace to remove moisture/solvent at 120˚C for 5 h and grinded until a uniform powder is obtained. Samples were subsequently annealed at 800˚C for 8 h by grinding and converting to a homogenous powder, a schematic diagram with proposed spinal structure as illustrated in Fig. 1. After annealing, samples were grinded for 1 h for fine powder achievement and then shifted to the specimen holders or air-tight bottles for examination.

The material was analyzed for structural, dielectric, and ultraviolet properties by X-ray diffraction (XRD), Scanning Electron Microscopy (SEM), Fourier transform infrared radiation (FTIR), Mössbauer spectroscopy, impedance analysis, and UV-Vis spectroscopy. For impedance analysis, 10 g of powder is compressed under 1 MPa for one minute to make pellets and sintered at 600 °C for 6 h to remove moisture. Silver paint was used to apply contacts subsequently, and samples were connected to the sample holder for analysis. Leads were checked carefully to ensure the absence of any irrelevant resistive or capacitive coupling agent in the measured frequency range. Completely automatic WINDETA software was used for interfacing the experimental setup of the analyzer to the computer and for data acquisition. All the graphs in this study were plotted using Origin (pro-2024) software, whereas, for schematic illustrations, Microsoft PowerPoint (2016) was used.

## Results and discussions

### Structural analysis

**Fig. 2:**
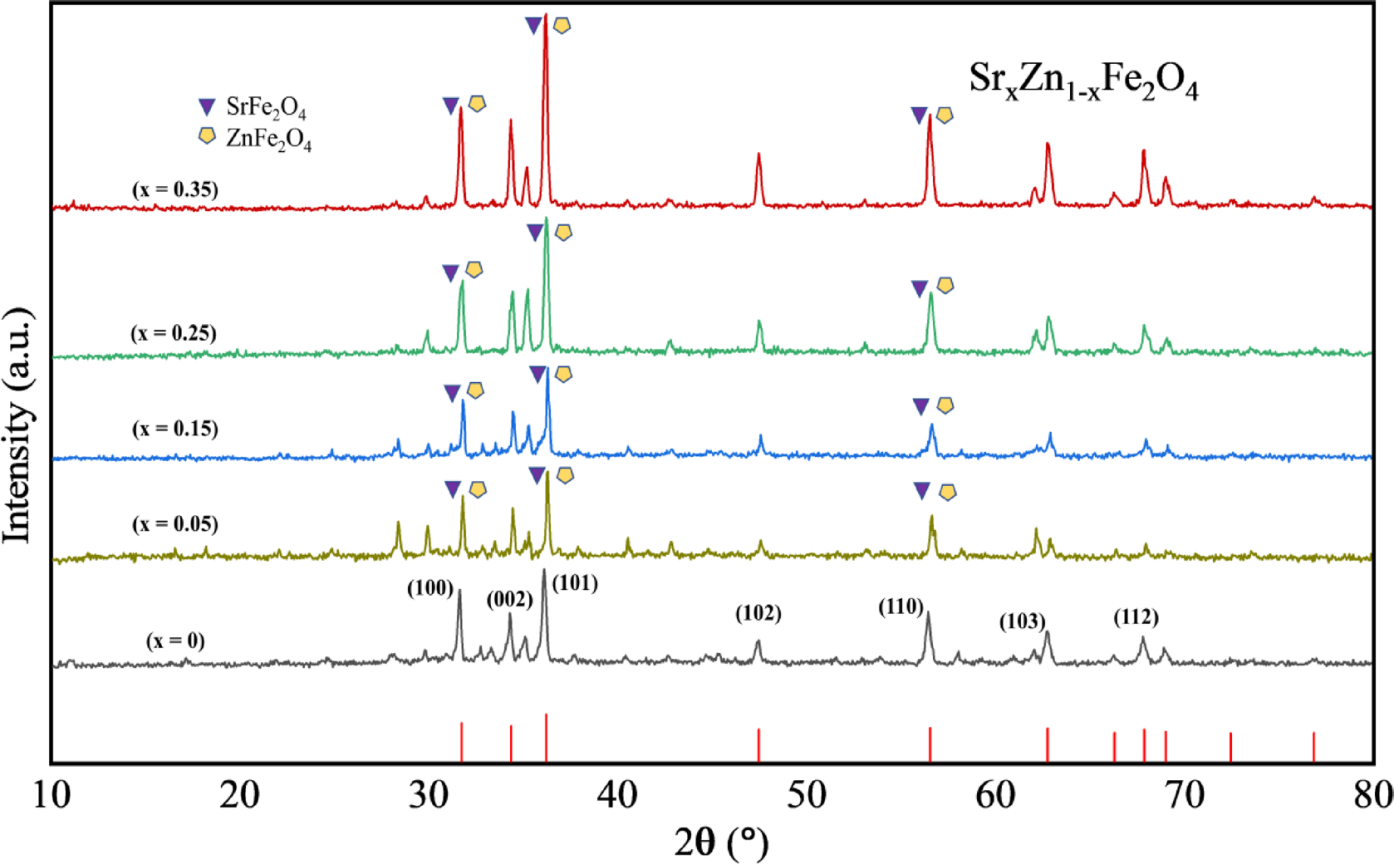
Crystallographic analysis using X-ray diffraction (XRD) pattern of Sr_x_Zn_1-x_Fe_2_O_4_.

Crystallography and phase examination of the powder sample were obtained using the X-ray diffraction (XRD) process. The angular range of 10º ≤ 2θ ≤ 80º with a step size of 0.05º and Cu-Kα radiations was allowed to fall on the sample with fixed wavelength (λ = 1.5418 Å). The high-resolution XRD patterns of annealed powder Sr_x_Zn_1−x_Fe_2_O_4_, where 0 ≤ x ≤ 0.35 are shown in Fig. 2. Recorded peaks were analyzed and found consistent with the JCPDS# 04–007–6616 for pure zinc ferrite (ZnFe) and JCPDS# 82–1049 and 22–1012 for SZF, phases of our study were present in these cards, confirming the single-phase cubical structure with uniform distribution of strontium in zinc ferrite nano-powder, with space group of Fd-3 m^21,22^. The subtle shift of diffraction peaks toward higher 2θ values in Fig. 2 is attributed to the incorporation of strontium and the simultaneous reduction in zinc content. With increasing Sr concentration, a notable change is observed in the intensity of specific peaks. Contrary to initial interpretation, the peak at ~ 36.14° becomes more pronounced as the Sr doping level rises, indicating improved crystallinity and possible lattice distortion due to ionic substitution. This trend confirms the progressive substitution of Zn²⁺ ions (0.74 Å) with larger Sr²⁺ ions (1.18 Å), which influences the crystal structure and enhances peak definition. The XRD patterns in Fig. 2 exhibit a gradual shift of diffraction peaks toward higher 2θ values with increasing Sr content, indicating a net lattice contraction. This counterintuitive behavior, considering the larger ionic radius of Sr²⁺ compared to Zn²⁺, can be attributed to defect-induced lattice distortions, internal compressive strains, and possible non-stoichiometry arising from Sr substitution. These factors collectively contribute to the observed decrease in lattice parameters, aligning with similar observations in doped spinel ferrite systems, as referenced in Table 1. It is theoretically anticipated that the lattice will expand and the unit cell volume will rise when Zn²⁺ ions (0.74 Å) are replaced with larger Sr²⁺ ions (1.18 Å). However, as Table 1 illustrates, the diffraction peak at about 36° gradually moves to higher 2θ values as the Sr concentration increases (36.14° → 36.50°), which is consistent with a reduction in the lattice parameter a (3.512 → 3.479 Å) and d-spacing (2.483 → 2.460 Å). The cation redistribution between tetrahedral and octahedral locations, oxygen non-stoichiometry, and defect-mediated compressive strains are responsible for this unanticipated lattice contraction, which offsets the size effect of Sr²⁺ ions. Internal lattice distortions caused by Sr incorporation are confirmed by the computed unit cell volumes and micro-strain values, which show a complicated interaction between ionic size, structural flaws, and cation site occupancy in the spinel framework. The prominent diffraction peaks correspond to 2θ values of (220), (110), (311), (400), (511), (440), (620), and (533), consistent with the standard spinel cubic phase with space group of Fd-3 m, reaffirming the formation of the desired crystalline structure^21^. The creation of the spinel cubic structure is confirmed by the XRD patterns of Sr-substituted ZnFeO₄ ferrites. Samples with a greater Sr concentration (x ≥ 0.35) show minor additional peaks, which suggests that a secondary phase, most likely SrFeO₄, formed as a result of Sr surpassing the percolation limit in the host ZnFeO₄ lattice. The intensity of the main peaks indicates that the primary spinel phase is still dominant^21^. The ionic characteristics of Sr²⁺ in the ZnFe₂O₄ spinel lattice explain its site preference. e³⁺ ions occupy octahedral (B) sites, while Zn²⁺ ions occupy tetrahedral (A) sites. Due to its higher ionic radius, Sr²⁺ replaces Zn²⁺ at lower concentrations at A-sites. As Sr concentration increases (x >0.25), partial migration of Sr²⁺ towards B-sites occurs, limiting lattice strain and preserving charge equilibrium. Redistribution generates defect-mediated compressive strain, as demonstrated by XRD peak shifts and poorer lattice characteristics. Sr inclusion regulates spinel framework lattice deformation and cation rearrangement, modulating structural and dielectric response^28,50^.

The SrₓZn₁₋ₓFe₂O₄ ferrite series (x = 0.00 to 0.35, a-e), represented by samples a ~ e, exhibits Rietveld refinement patterns that validate the successful crystallization into a single-phase cubic spinel structure indexed to Franklinite (space group Fd-3 m), as previously discussed^22^.

**Table 1:** Maximum intensity calculation for (101), justifying peak shift towards the right.

Sr Doping (x)	2θ (°)	d (Å)	a (Å)
0.00	36.14	2.483	3.512
0.05	36.24	2.477	3.503
0.15	36.32	2.472	3.495
0.25	36.42	2.465	3.486
0.35	36.50	2.460	3.479

### Rietveld refinement

**Fig. 3:**
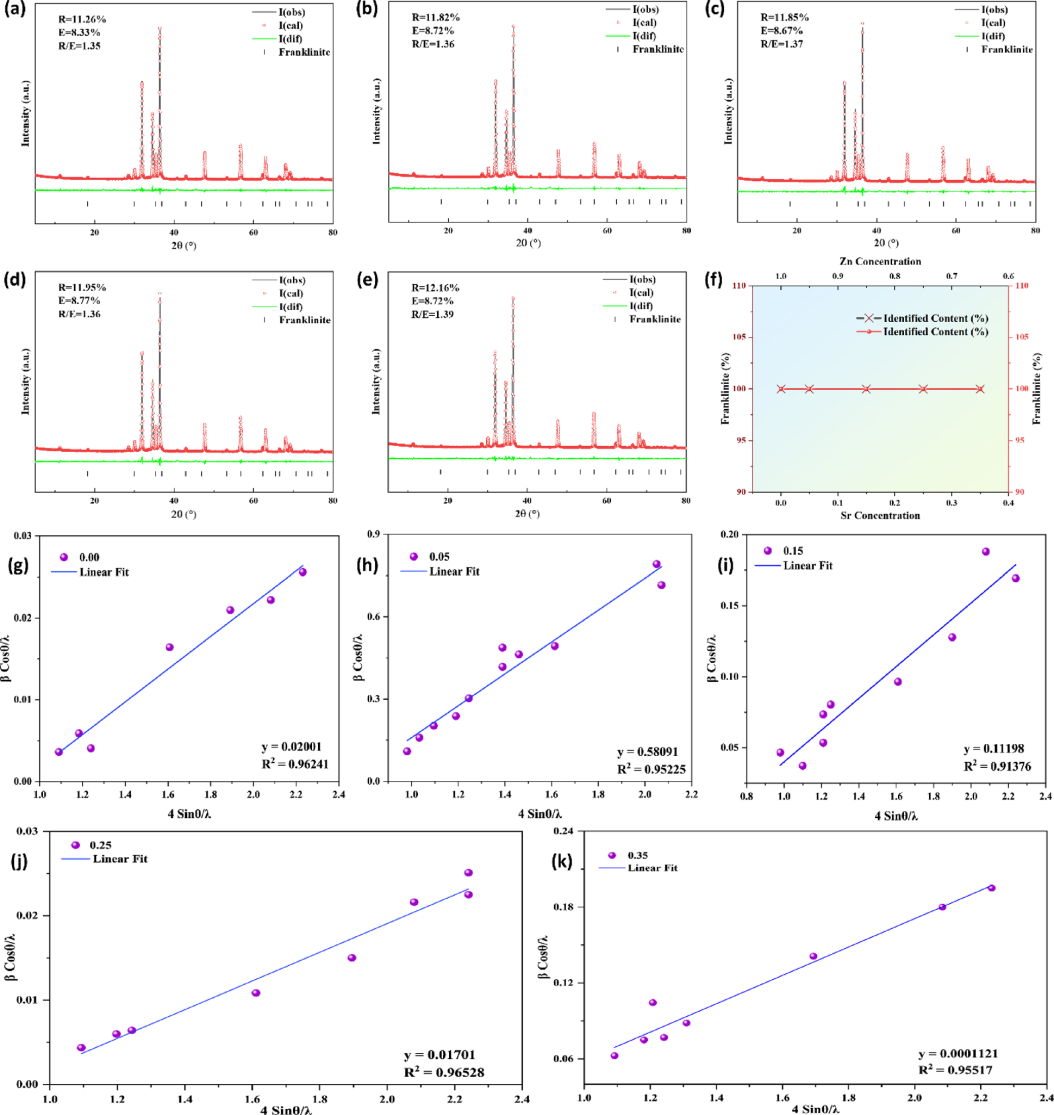
Rietveld Refined and Williamson-Hall from X-ray diffraction (XRD) pattern of Sr_x_Zn_1−x_Fe_2_O_4_ are displayed. The improved XRD patterns for x = 0.00–0.35.00.35 are displayed in (a-e), demonstrating excellent agreement between the calculated and observed profiles as well as phase purity and structural regularity. (f) Sr concentration variation across the doping series. (g-k) show the matching W-H plots (βcosθ vs. 4sinθ), which are used to calculate the lattice strain and mean crystallite size. The strong linearity (R^2^ = 0.94–0.96) confirms the homogeneous strain–size relationship, while the positive slopes show tensile micro-strain brought on by Sr replacement.

The strong correlation between the observed and predicted XRD profiles, along with negligible residuals in the difference plots, underscores the precision of the structural model employed for refinement. The refinement parameters, R-factors between 11.26% and 12.16% and goodness-of-fit (χ²) values near 1.35 are within the acceptable range, signifying a statistically sound and reproducible fit across all doping concentrations. The lack of peak broadening, amorphous humps, or secondary phase reflections indicates that Sr²⁺ ions integrated into the Zn²⁺ lattice positions without causing phase segregation or structural distortion. Figure 3**(a-e)** represents the Rietveld refinement patterns of x = 0, 0.05, 0.15, 0.25, and 0.35, labeled as a, b, c, d, and e, respectively. The Fig. 3f also presents the doping concentrations of Sr and Zn, respectively. The uniformity observed in the doping series indicates that Sr substitution maintains the integrity of the host spinel structure and enhances phase stability under the specified synthesis conditions. The resulting structural integrity establishes a robust basis for linking the material’s microstructural and dielectric properties to its compositional variations.

Employing the uniform deformation model (UDM), the Williamson-Hall (W-H) approach was used to further clarify the contributions of crystallite size and lattice strain to X-ray peak broadening. For every Sr doping concentration (x = 0, 0.05, 0.15, 0.25, and 0.35), the βCosθ against 4Sinθ graphs are displayed in Fig. 3(g-k). The slope represents the micro-strain (ε) formed within the nanocrystals, and the y-intercept represents the instrumental broadening related to crystallite size in each linearly fitted dataset. The dependability of the W-H method in measuring the strain-size interaction is confirmed by a steady linear trend with high regression coefficients (R² = 0.94–0.96). All compositions exhibit structural homogeneity, as confirmed by the close agreement between the computed crystallite sizes and those derived from the Scherrer equation. The presence of tensile lattice strain is indicated by the positive slope in each panel, which progressively rises with Sr inclusion. This suggests that the oxygen coordination environment is slightly distorted as a result of the ionic radius mismatch between Sr²⁺ (1.18 Å) and Zn²⁺ (0.74 Å). According to the Rietveld refinement results, which showed phase purity and structural coherence, Sr doping does not appear to cause considerable internal strain or defect clustering, as indicated by the comparatively tiny values of both slope and intercept^21–27,50^. The crystalline domains are thus confirmed to be well ordered by the W-H analysis, and the observed widening is mainly caused by nanoscale crystallite effects rather than lattice defects.

**Fig. 4:**
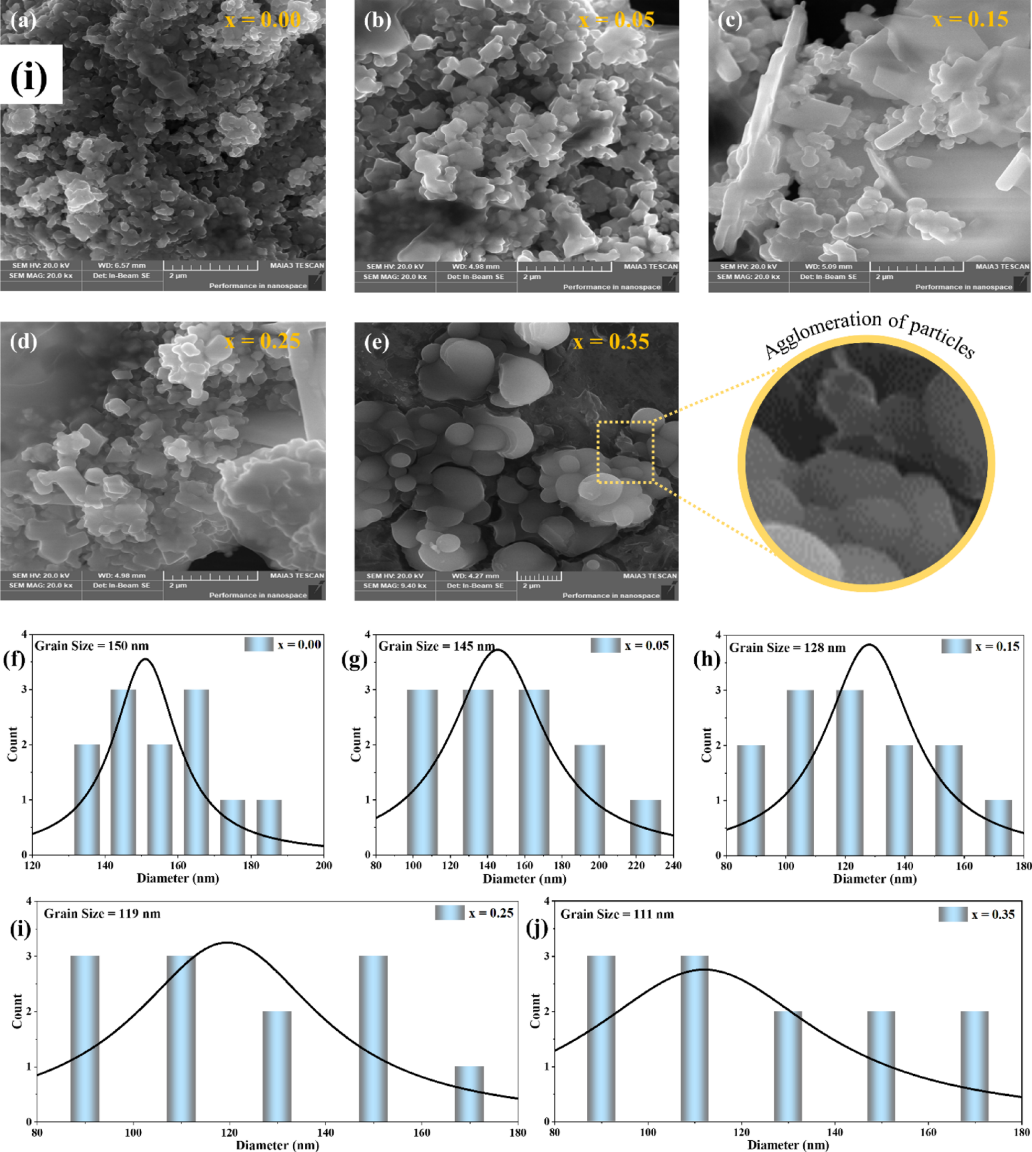

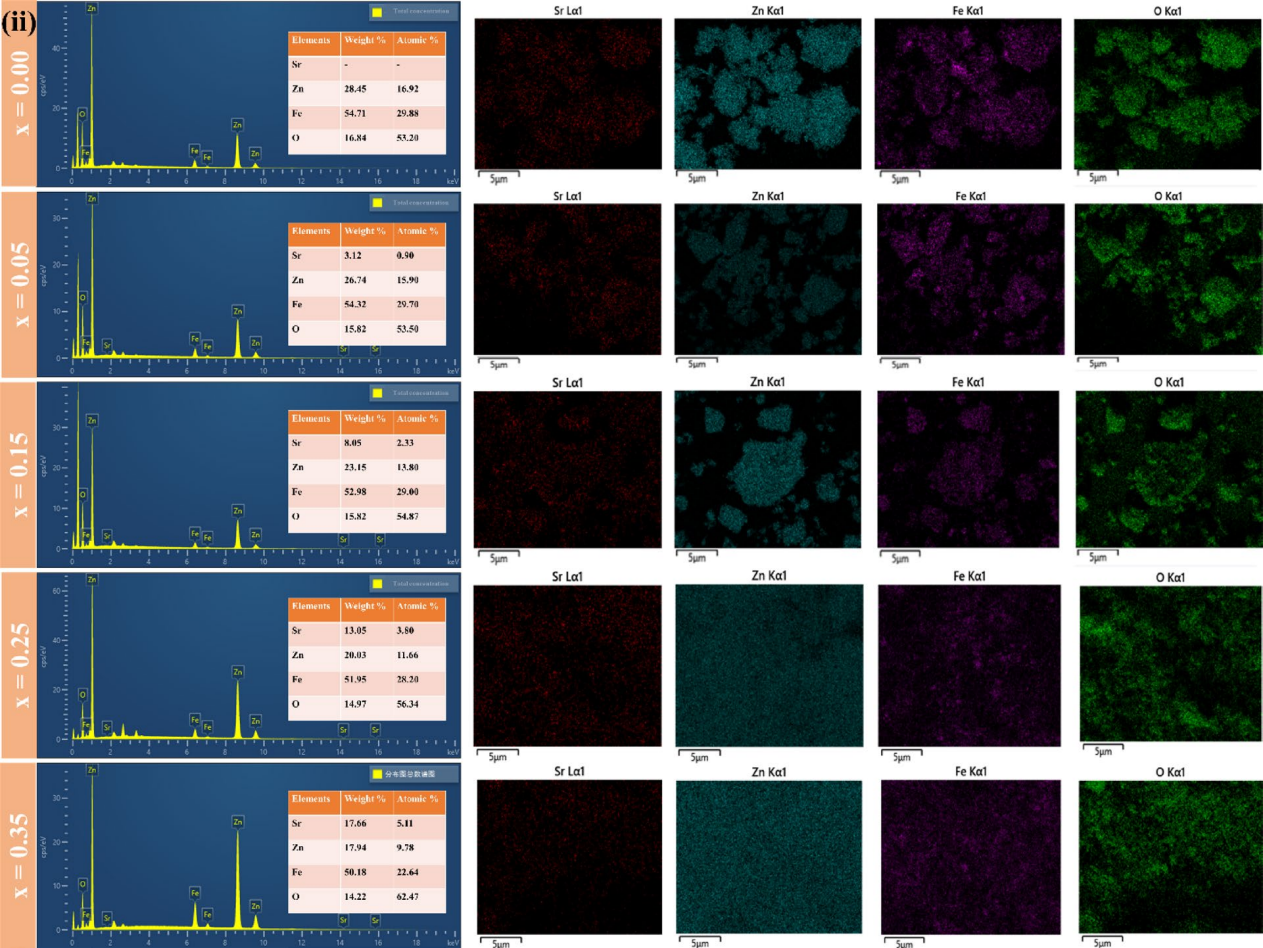
Visualization and representation for agglomeration of SZF particles. (i) The qualitative representation of FESEM micrographs of SrₓZn₁₋ₓFe₂O₄, (x = 0, 0.05, 0.15, 0.25, 0.35) with their quantitative data using ImageJ software for particle size distribution. (ii) The elemental mapping and EDX spectra of SrₓZn₁₋ₓFe₂O₄ (0 < x ≤ 0.35) verify the presence and even distribution of Sr, Zn, Fe, and O. Elemental maps confirm uniform composition without phase segregation, but EDX data demonstrate systematic Sr incorporation with increasing x.

Surface morphology was examined via Field Emission Scanning Electron Microscopy (FESEM, TESCAN MAIA3). The micrographs shown in Fig. 4**(i)(a-e)** display the FESEM images of the synthesized Sr_x_Zn_1−x_Fe_2_O_4_ samples corresponding to x = 0, 0.05, 0.15, 0.25, and 0.35, respectively. These high-magnification images show a distribution of nanoparticles across all samples, although the particle sizes are not uniform, and some agglomeration is observed, particularly in the sample with x = 0. This variation suggests that further optimization could benefit the synthesis process for better particle size control. The particle distribution appears non-homogeneous in the sample with x = 0, with agglomerated crystals ranging from 1 to 5 μm. However, excellent particle-to-particle contact is observed, suggesting strong interfacial bonding between the particles. This close interaction indicates potential for enhanced electron transport, which makes the material promising for optical applications, which can be further explored through Ultraviolet-Visible analysis.

Figure 4**(i)(f-j)** presents the statistical information of particle size for Sr_x_Zn_1−x_Fe_2_O_4_. As the Sr content increases to x = 0.05, the particle morphology exhibits less agglomeration, with crystal sizes in nm. For the sample with x = 0.15, the addition of Sr leads to a reduction in isolated voids, influenced by the ionic radii of Sr (1.18 Å), O (1.4 Å), Fe (0.645 Å), and Zn (0.74 Å). With further doping to x = 0.25 and 0.35, the gap reduction between grains becomes more pronounced, as the enlargement of strontium’s atomic radii increases the number of larger particles. In these samples, the crystal diameters approximate 0.5 μm, representing a significant improvement in particle distribution. Figure 4**(ii)**. Elemental mapping and energy-dispersive X-ray spectroscopy (EDX) spectra of SrₓZn₁₋ₓFe₂O₄ (0 ≤ x ≤ 0.35) samples verify the elemental composition and uniform distribution of constituent elements. The existence and consistent rise in Sr peaks with increasing Sr concentration (x = 0.00 to 0.35), as shown in the EDX spectra (left panels), confirm that Sr was successfully substituted into the Zn-ferrite lattice. The atomic percentages and associated weights of Sr, Zn, Fe, and O for each composition are shown in quantitative data tables (insets). All samples exhibit homogenous incorporation of Sr ions without discernible phase segregation, as shown by the elemental mapping pictures (right panels), which show uniform distribution of Sr (red), Zn (cyan), Fe (violet), and O (green).

### Functional group identification

**Fig. 5:**
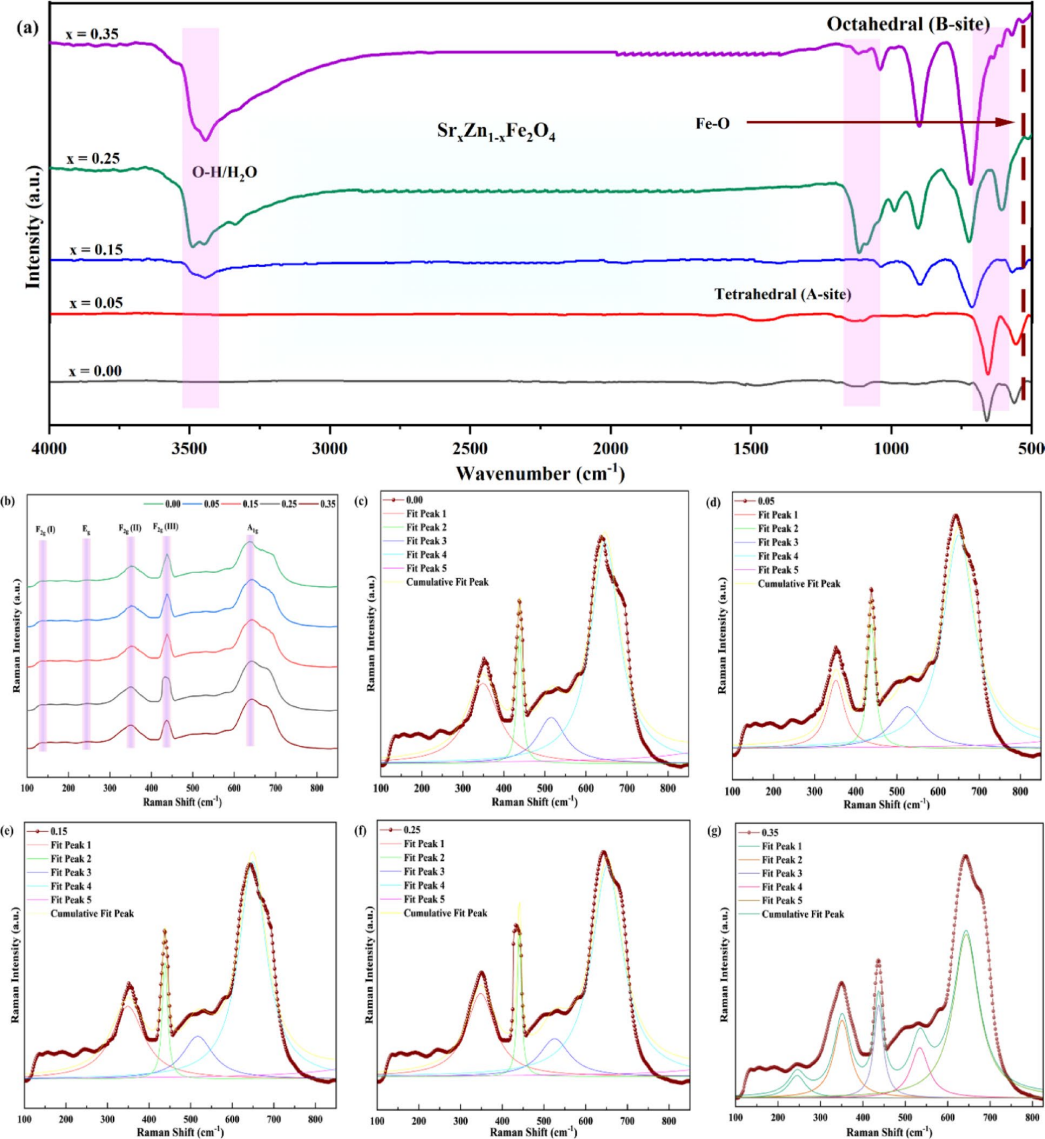
Functional group identification is shown. (**a**) The spinel ferrite structure is confirmed by the typical Fe-O and Zn-O stretching vibrations seen in the FTIR spectra of SrₓZn₁₋ₓFe₂O₄ (0.00 ≤ x ≤ 0.35). (b-g) Lorentzian-fitted profiles and Raman spectra for varying Sr concentrations. Both symmetric and asymmetric oxygen vibrations within the Fe-O and Zn-O bonds are represented by the observed A₁g and F₂g modes. Phase purity and structural stability throughout compositions are confirmed by the preservation of all distinctive Raman bands. In contrast, slight redshifts and intensity changes with increasing Sr²⁺ content suggest lattice distortion and cation redistribution.

Functional group identification was carried out by scanning the samples prepared by the coprecipitation method to localize the position of ions. FTIR analysis was performed in the IR region 4000–400 cm^− 1^, confirming the prepared spinel zinc ferrite formation as shown in Fig. 5**(a)**. It is found that the region 400–600 cm^− 1^ is specifically for strontium hexaferrite due to the widening of the octahedral and tetrahedral vibration of metallic oxygen. It affirms that these regions indicate the presence of SZF material in composite form. The band associated with the Fe-O (iron-oxygen) bond is located at 570 cm^− 1^ within a tetrahedral site, indicating a precise arrangement of iron and oxygen atoms. The region 600–1570 cm^− 1^ shows the asymmetric and symmetric stretching of the O-Sr bond, and 720–725 cm^− 1^ peaks show Zn-O bending vibrations, respectively. As the concentration of strontium increases in the composite, some interesting phenomena are observed, including a shift in the band, which confirms the strontium doping in zinc ferrites and enhances the modification of local electronic environments. Strontium and zinc are divalent ions, which is why Sr-ion and iron partially occupy both the tetrahedral and octahedral sites while Zn-ion inhabits the tetrahedral site. The FTIR spectra offered crucial insights into the structure and composition of Sr_x_Zn_1−x_Fe_2_O₄ composites. Through detailed analysis of bands, scientists can gain a deeper understanding of the material properties, paving the way for their utilization in a variety of spinel ferrite applications. A pristine spinel structure is confirmed to have formed by the FTIR spectra of SrₓZn₁₋ₓFe₂O₄ (0 < x ≤ 0.35). The tetrahedral (A-site) and octahedral (B-site) Fe-O stretching vibrations are responsible for the significant absorption bands located around 738–722 cm⁻¹ and 587–560 cm⁻¹, respectively. A progressive redshift in both bands as the Sr^2+^ content rises verifies that the ionic substitution was successful, leading to local symmetry distortion and lattice expansion. The observed Fe-O stretching vibration at ~ 570 cm⁻¹, attributed to the tetrahedral site, confirms the structural integrity of the spinel ferrite lattice; its shift with increasing Sr content reflects cationic substitution and local lattice distortion, which can modulate magnetic ordering and dielectric response in SrₓZn₁₋ₓFe₂O₄ composites^21^. The samples are monophasic, as evidenced by the lack of any distinctive peaks that fit the M-type strontium hexaferrite (~ 600 cm⁻¹). By eliminating overlapping peaks and enabling accurate wavenumber identification, deconvolution analysis reinforces these assignments even more. The higher the Sr content, the redshift (shift to lower wavenumbers) occurs in all three bands. This ongoing transition is referred to in Table 2 as attributed to: Lattice expansion brought on by the bigger Sr²⁺ (1.18 Å) replacing the smaller Zn²⁺ (0.74 Å). Elongation of the bond length results in weaker Fe-O bonds and a lower vibrational frequency. The extra Fe-O mode (~ 470–510 cm⁻¹) is probably caused by changed local coordination because of Sr substitution or Fe-O-Fe links in a deformed octahedral. The SrₓZn₁₋ₓFe₂O₄ (0.00 ≤ x ≤ 0.35) samples’ vibrational Raman spectra are shown in Fig. 5**(b-g)**. The spinel ferrite structure is represented by distinct Raman active modes, mainly A₁g and F₂g. These modes come from the stretching vibrations of oxygen atoms at tetrahedral and octahedral sites connected to Fe-O and Zn-O bonds, respectively, which are symmetric (A₁g) and asymmetric (F₂g). In order to precisely assess the lattice vibrational behavior and resolve overlapping vibration bands, the spectra were deconvoluted using Lorentzian line fitting. The validity of the deconvolution and the phase homogeneity are confirmed by the excellent agreement between the fitted curves and the experimental spectra. Progressive Sr²⁺ incorporation causes a discernible shift of the A₁g and F₂g peaks toward lower wavenumbers, suggesting that lattice expansion and the replacement of smaller Zn²⁺ ions (0.74 Å) with bigger Sr²⁺ ions (1.18 Å) soften the Fe-O stretching vibrations. This difference in ionic radius causes local lattice deformation and micro-strain, which alters the force constant of metal-oxygen bonding. This leads to a partial shift of Fe^2+^ ions from octahedral (B) to tetrahedral (A) sites, which affects local symmetry and cationic ordering. In accordance with the observed minor broadening of diffraction peaks in XRD analysis, the decrease in Raman peak strength at greater Sr content also indicates a weakening of short-range ordering within the oxygen sublattice^31–38^. Despite these compositional changes, the distinctive A₁g and F₂g modes are preserved at all doping concentrations, indicating that the cubic spinel structure is unaltered and no secondary phase has formed. All things considered, Raman analysis supports the FTIR results by confirming the changes in site occupancy, phase purity, and structural integrity brought on by Sr substitution in ZnFeO₄. The cation redistribution mechanism and the evolution of lattice strain with increasing Sr concentration are both significantly influenced by these tiny phonon changes.

**Table 2:** Shifting in bands with increasing Sr concentration.

Sr doping (x)	Tetrahedral a-site (cm⁻¹)	Octahedral b-site (cm⁻¹)	Fe-o (cm⁻¹)
0.00	738	587	510
0.05	734	582	506
0.15	729	575	500
0.25	726	568	494
0.35	722	560	488

### Optical absorption and band gap identification of SZF

**Fig. 6:**
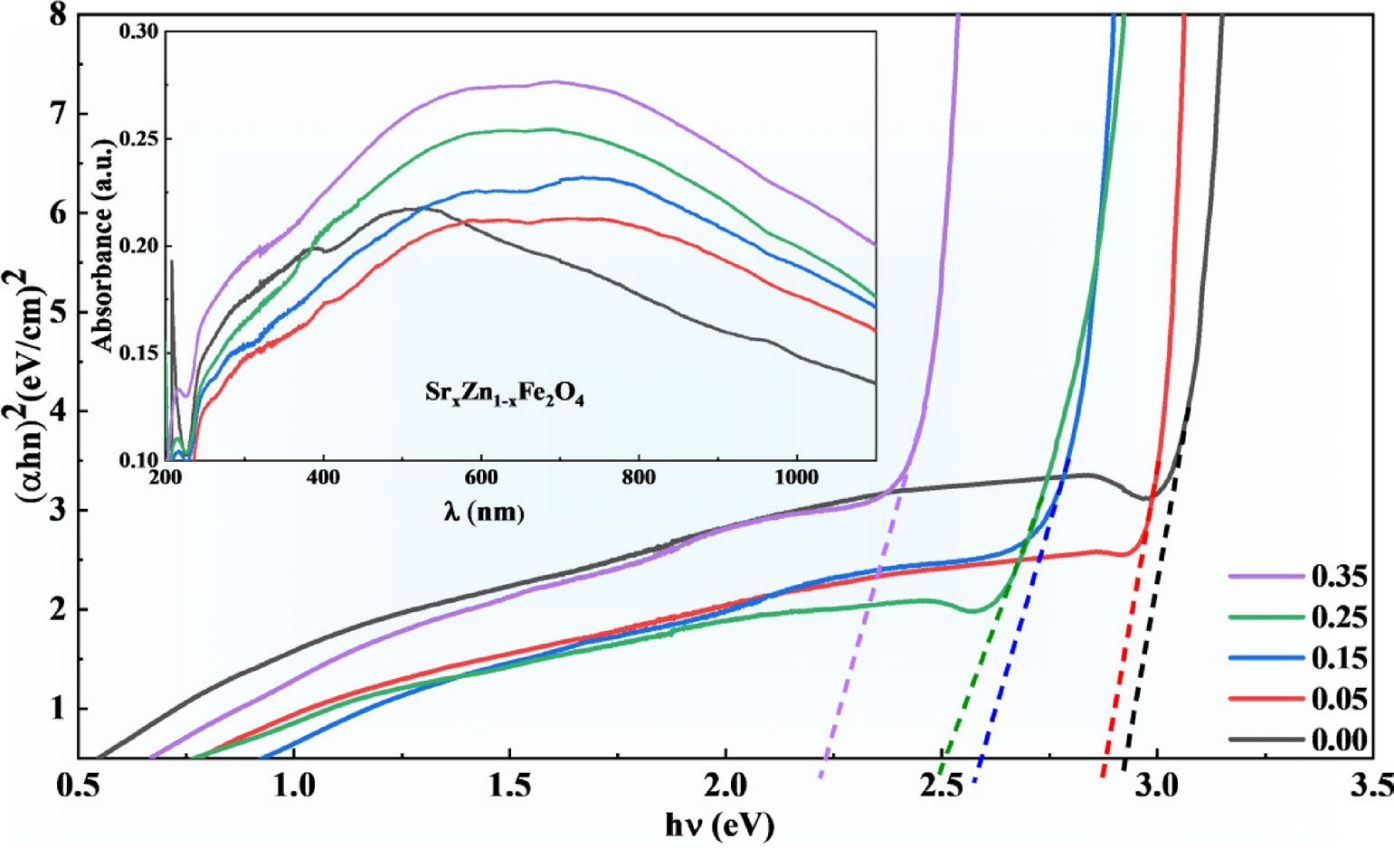
Tauc plots of Sr_x_Zn_1−x_Fe_2_O_4_​ (absorption vs. photon energy), the inset shows absorbance across various values of wavelength for the ultraviolet and visible regions.

UV-Vis absorption spectra of Sr_x_Zn_1−x_Fe_2_O_4_, depicted in Fig. 6, indicate that an increased strontium doping ratio improves the material’s absorption capacity, which implicitly points towards good behavior towards application. In SZF, the complete absorption array was fine in the UV region. A pure sample, with zero concentration of strontium in zinc ferrite, is also studied so that the effect of strontium doping can be seen for semiconductor and other optical applications using a Tauc plot, projected by the Tauc-Lorentz relation;

2 $$\:\begin{array}{c}{\left(\alpha\:h\upsilon\:\right)}^{n}\:=\:B\left(h\upsilon\:\:-\:{E}_{g}\right)\end{array}$$

Where α, hυ, E_g_, B, absorbance, photon energy, and optical band gap are constantly associated with the samples, *n* = 2, and n = ½ for direct and indirect transitions. The Sr_x_Zn_1−x_Fe_2_O_4_ sample’s optical band gap (E_g_) was calculated using the Tauc relation (Eq. 2). Following the usual procedure, *n* = 1/2 was used in the Tauc plot analysis for all compositions because ZnFeO₄ is known to show indirect permitted electronic transitions. The shift in the E_g_ towards higher energies with increasing Sr doping is primarily due to the larger ionic radii of Sr, which alter the material’s electronic structure, rather than focusing solely on the average E_g_. Observing how Sr doping influences the band gap across different concentrations is more insightful. At x = 0.05, a noticeable increase in Eg is observed compared to the undoped sample (x = 0), suggesting a subtle yet significant modification in the electronic properties. As the Sr concentration increases to x = 0.15 and higher, the band gap continues to gradual narrowing of the optical band gap (E_g_). In addition, the Tauc plots, constructed using the Tauc-Lorentz relation, show that the undoped sample (x = 0) has the largest E_g_, which gradually decreases for the other values of doping, highlighting the progressive effect of Sr on the material’s electronic structure. These results demonstrate the impact of Sr doping and confirm that the material’s E_g_ falls within the optimal semiconducting range of 2–3 eV, which is desirable for optoelectronic and semiconducting applications. The enhancement in E_g_, evidenced by the shift in the (αhυ)^n^ curve, reflects a marked improvement in the semiconducting properties, making the material more suitable for advanced technological applications. For real-world uses including photocatalysis in visible light, solar energy conversion, photodetection, and spintronic devices, where semiconductors with adjustable Eg values in this window are greatly desired, this band gap range (2.2–2.8 eV) is very pertinent. These findings corroborate and extend previous studies, showcasing the potential of Sr doping to fine-tune the material’s electronic behavior for targeted applications^21–23^.

### Surface chemistry and oxidation state

**Fig. 7:**
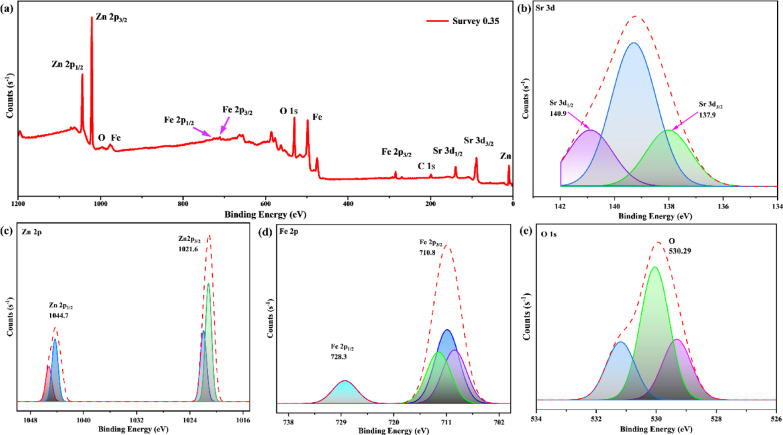
X-ray Photoelectron Spectroscopy (XPS) spectra of the optimized Sr₀.₃₅Zn₀.₆₅Fe₂O₄ sample showing (**a**) survey scan confirming elemental composition, and high-resolution spectra of (**b**) Sr 3 d, (c) Zn 2p, (d) Fe 2p, and (e) O 1 s core levels. The deconvoluted peaks reveal Sr²⁺ substitution within the spinel lattice, the coexistence of Fe³⁺/Fe²⁺ states, and oxygen vacancy formation associated with charge compensation.

The optimized Sr-doped ZnFe₂O₄ sample (x = 0.35) was analyzed using XPS after optical examination to determine its surface composition and oxidation states. The best-performing composition’s comprehensive investigation revealed the electronic structure and confirmed Sr, Zn, Fe, and O’s chemical states. The XPS study, Fig. 7, confirms the effective substitution of Sr²⁺ into the ZnFe₂O₄ lattice, including charge compensation and oxygen vacancy production mechanisms. The presence of Sr, Zn, Fe, and O without impurities is confirmed by XPS analysis of Sr₀.₃₅Zn₀.₆₅Fe₂O₄, confirming good sample purity. Its substitution into the spinel lattice is confirmed by the Sr 3 d peaks at 133.8 and 135.4 eV, which correspond to Sr²⁺. Zn²⁺ is found in tetrahedral positions, as indicated by Zn 2p peaks at 1021.6 and 1044.7 eV, demonstrating that Sr inclusion does not change Zn valence. The coexistence of Fe³⁺ and Fe²⁺ states are revealed by the Fe 2p spectrum, which displays peaks at 710.9 and 724.5 eV with a weak satellite at 718.6 eV. The creation of oxygen vacancies as a result of Sr²⁺ replacement and charge compensation are the cause of the small increase in Fe²⁺ content. Lattice oxygen and surface-adsorbed oxygen species, respectively, are represented by components in the O 1 s spectra at 530.2 and 531.6 eV, demonstrating the presence of oxygen-deficient locations. The combined findings from previous works confirm that Sr²⁺ is effectively integrated into the ZnFe₂O₄ lattice, preserving a mixed-valence Fe environment and generating oxygen vacancies that are in line with those found in Sr-doped spinel ferrites [52 ].

### Hyperfine interaction study using Mössbauer spectroscopy

**Fig. 8:**
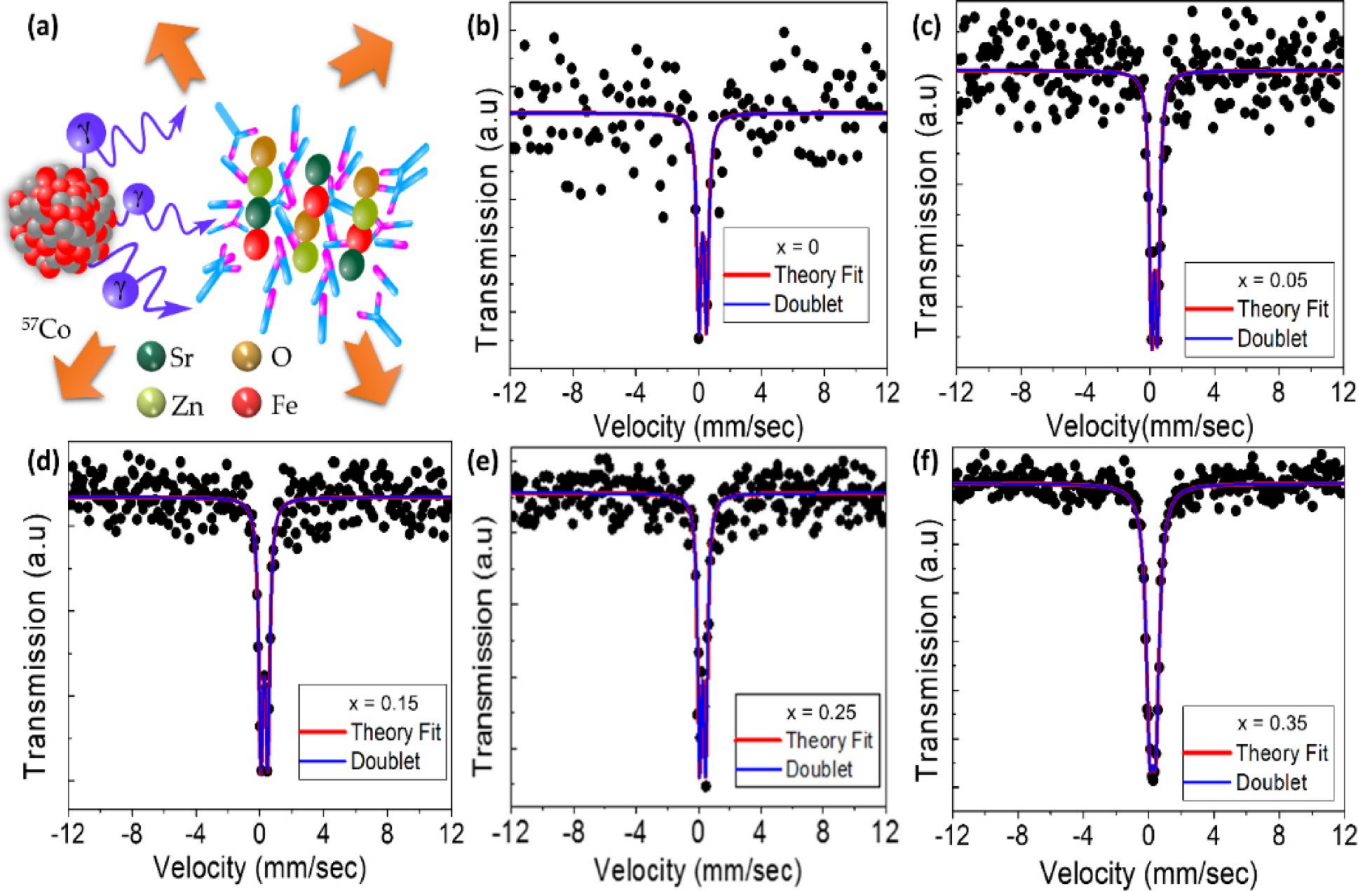
Mössbauer spectra for Sr_x_Zn_1−x_Fe_2_O_4_. (**a**) Schematic illustration of Mössbauer spectroscopy; whereas (**b**), (**c**), (**d**), (**e**), and (**f**) represent observed behavior of x = 0, 0.05, 0.15, 0.25, and 0.35.

Mössbauer spectra of Sr_x_Zn_1−x_Fe_2_O_4_ with (0 ≤ x ≤ 0.35) specimens recorded at ambient temperature. Hyperfine Parameters of isomer shift (IS), quadrupole shift (QS), and width were obtained during this test, and adjustment was compared to a 25 μm, dense specimen of high-purity alpha iron, as represented in Fig. 8**(a)**. The quantitative data of the Mössbauer bands of magnetic apparatuses have been tailored using the Lorentz line setting to access a couple of connections with a minimum ϰ2 value of ~ 1.0. A processor-fitted Lorentzian curve is shown as a solid line; the obtained parameters are shown in Table 3.

In Fig. 8, the magnetic properties of SZF using Mössbauer spectroscopy reveal a Sr-dependent transition towards superparamagnetic from paramagnetic behavior. As of Table 3, all samples displayed quadrupole-split doublets with isomer shifts (δ) between 0.35 and 0.38 mms^− 1^, indicating high-spin Fe^3+^ ions occupying both (A-site) tetrahedral and (B-site) octahedral positions with a spinel lattice. The quadrupole splitting parameter (Δ) exhibited a systematic decrease from 0.49 mms^− 1^ (x = 0) to 0.37 mms^− 1^ (x = 0.35), suggesting a reduction in lattice distortion as Sr^2+^ ions substitute Zn^2+^ at the A-site. This trend leads to near-cubic symmetry, which reduces magnetic anisotropy and promotes paramagnetic relaxation.

Notably, the line width (Γ) remained constant at 0.32 mms^− 1^ for x = 0 - 0.15, reflecting uniform Fe3 + environments at lower doping concentrations. However, a rapid increase in Γ to 0.53 mms^− 1^ for x = 0.35 indicates the onset of paramagnetic conduct, characterized by a distribution of hyperfine fields due to Sr-induced lattice disorder. This widens the superparamagnetic threshold. The absence of a resolved sextet in the spectra further chains a weak active magnetic field (H_eff_ ˂ 5 T), ruling out conventional ferromagnetism.

Strontium doping in zinc ferrite influences the magnetic interactions through two primary mechanisms. First, Sr^2+^ ions replace non-magnetic Zn^2+^ at the A-site, facilitating Fe^3+^ migration to octahedral B-sites, where enhanced Fe^3+^-O^2−^ orbital overlap reduces local magnetic moments. This is evident by the isomer shift trend: δ increases from 0.36 mms-1 (x = 0, A-site dominance) to 0.38 mms-1 (x 0.05, B-site dominance), reflecting stronger covalent bonding at octahedral positions. Second, calcination temperature significantly affects crystallite size; better-calcined samples exhibit reduced line broadening (e.g., Γ = 0.29 mms^− 1^ for x = 0.25), stabilizing the hyperfine environment. In this way, all samples were calcined at 800 °C for 6 h in air to promote crystallinity and phase purity. This thermal treatment has significantly influenced the grain growth, as evident from the reduced line width (Γ = 0.29 mm/s for x = 0.25), reflecting enhanced hyperfine homogeneity. Higher calcination temperature facilitates the elimination of surface defects and suppresses spin canting, which are known to broaden Mössbauer lines. Additionally, improved crystallite size leads to reduced superparamagnetic relaxation effects, sharpening the spectral features. This chains the detected trend of magnetic ordering stabilization with Sr substitution, particularly at modest doping levels.

The interplay between Sr concentration and magnetic behavior positions it as a versatile material for applications requiring low coercivity, such as high-frequency transformers or magnetic hyperthermia agents. The gradual increase in isomer shift (IS) from 0.36 mm/s to 0.38 mm/s with initial Sr substitution (x = 0 to 0.05) can be attributed to Fe³⁺ ions preferentially occupying more covalently bonded octahedral (B) sites, where the electron density at the nucleus is enhanced. This shift is supported by the relatively larger ionic radius of Sr²⁺ (1.18 Å) compared to Zn²⁺ (0.74 Å), which induces local structural distortion and enables Fe³⁺ redistribution. The stronger Fe-O orbital overlap in B-sites increases electron density at the Fe nucleus, accounting for the observed IS elevation. To depict Fe³⁺ ions in the tetrahedral (A) and octahedral (B) spinel sites, the room-temperature Mössbauer spectra of Sr_x_Zn_1–x_Fe_2_O_4_ (0 < x ≤ 0.35) were re-analyzed using two quadrupole doublets. With a smaller isomer shift (IS = 0.3–0.35 mm/s) attributed to Fe³⁺ at the A-sites, and a bigger isomer shift (IS ≈ 0.45–0.50 mm/s) ascribed to Fe³⁺ at the B-sites, we find that each spectrum is well matched by one doublet. These quadrupoles splitting (QS ~ 0.25 - 0.35 mm/s) and IS values show virtually symmetric local environments at both sites and are characteristic of Fe³⁺ in nanoscale spinel ferrites. The fitted relative areas of the two doublets exhibit a systematic change with varying Sr content: the area of the B-site (larger-IS) component decreases, while the area of the A-site (smaller-IS) component increases as x increases. With Sr doping, this pattern suggests a rise in cation inversion (more Fe³⁺ on A-sites). This behavior is in line with the bigger Sr²⁺ ion preferentially occupying octahedral (B) sites and pushing Zn²⁺ or Fe³⁺ into the A sites^21,50^. In conclusion, mixed A/B occupancy of Fe³⁺ is directly confirmed by the two-doublet Mössbauer analysis, which also lends credence to the idea that Sr substitution causes cation redistribution between the tetrahedral and octahedral sites.

**Table 3:** Mössbauer spectroscopic parameters for Sr_x_Zn_1-x_Fe_2_O_4_ nanoparticles.

Sr doping	Specimen ID	Quadrupole splitting	Isomer shift	Width
0	Sr_0_Zn_1_Fe_2_O_4_	0.49	0.36	0.32
0.05	Sr_0.05_Zn_0.95_Fe_2_O_4_	0.38	0.38	0.32
0.15	Sr_0.15_Zn_0.85_Fe_2_O_4_	0.42	0.37	0.32
0.25	Sr_0.25_Zn_0.75_Fe_2_O_4_	0.38	0.35	0.29
0.35	Sr_0.35_Zn_0.65_Fe_2_O_4_	0.37	0.35	0.53

### Impedance spectroscopy investigation for electronic applications

**Fig. 9:**
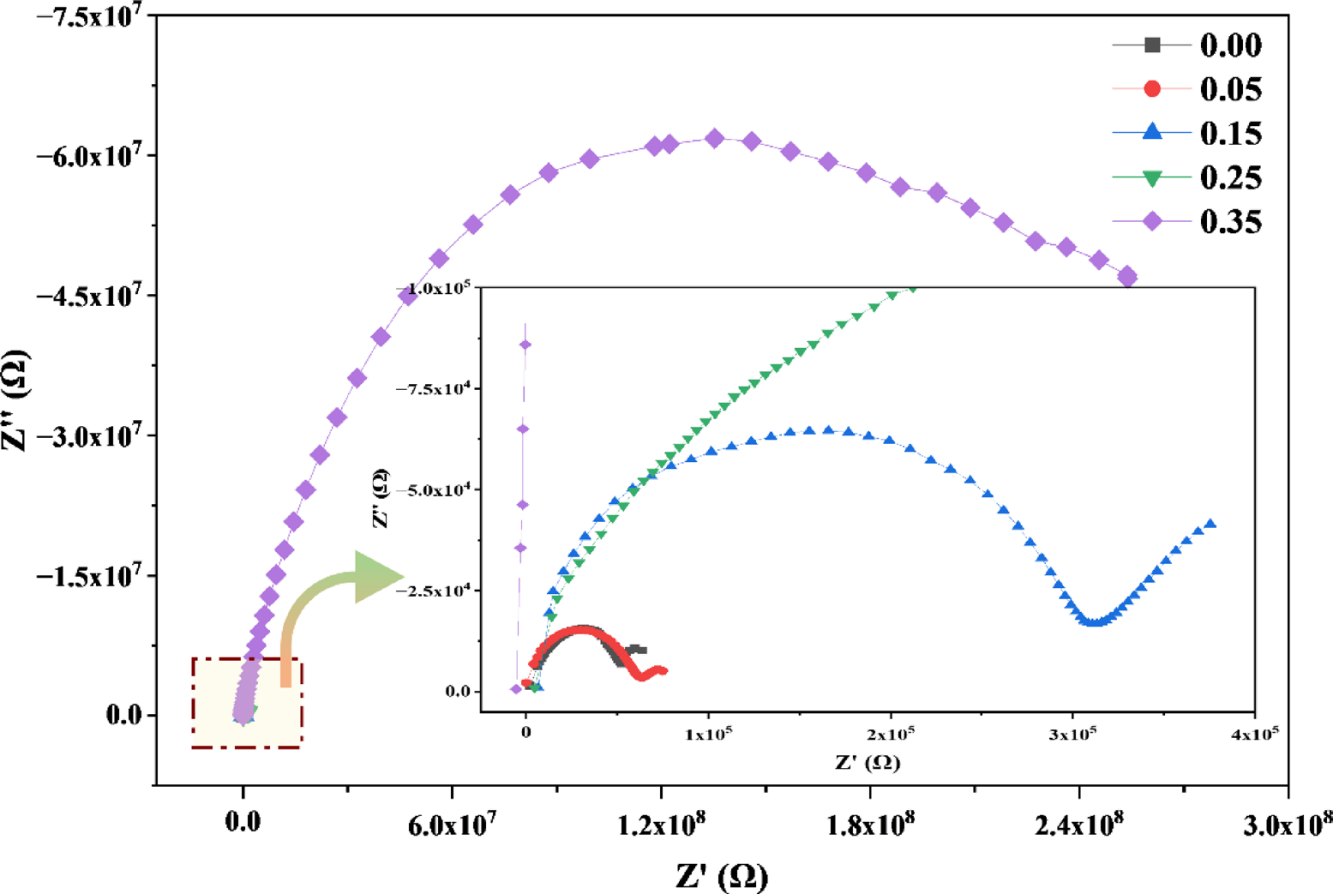
Frequency-dependent impedance plots (imaginary vs. real component) of Sr_x_Zn_1-x_Fe_2_O_4_ at room temperature, showing increasing capacitive behavior and non-Debye relaxation with Sr doping.

The application-based impedance study of Sr_x_Zn_1−x_Fe_2_O_4_ at ambient temperature, as shown in Fig. 9, ascertains that the material reflects a talented situation with an increasing doping ratio. In neighboring environments, the dominant phases and defects within the studied materials can lead to multiple relaxation processes. As a result, during the analysis of impedance (Z’’ vs. Z’ plots), it can be challenging to separate the opposing phases due to variations in polarization decay.

All the sample graphs exhibit a depressed semicircular arc, with centers located below the real axis, indicating a non-Debye relaxation behavior due to multiple time constants within the system^18,19^. Instead, the observed impedance response shows a continuous rise without forming arcs, indicating the dominance of interfacial polarization and grain boundary resistance. This behavior reflects a non-Debye type relaxation mechanism, likely caused by structural disorder, localized defect states, and limited charge carrier mobility introduced by doping. As a result, the system’s electrical response is best modeled using constant phase elements rather than ideal capacitive components, capturing its dispersive and non-ideal dielectric nature. This angle increases with higher concentrations. Additionally, all plots exhibit a low-frequency dispersion followed by a plateau region that starts from the same initial point. The dispersion region extends into a higher frequency range as the concentration increases. This result is consistent with previous work^24^.

### Dielectric features of SZF composites

**Fig. 10:**
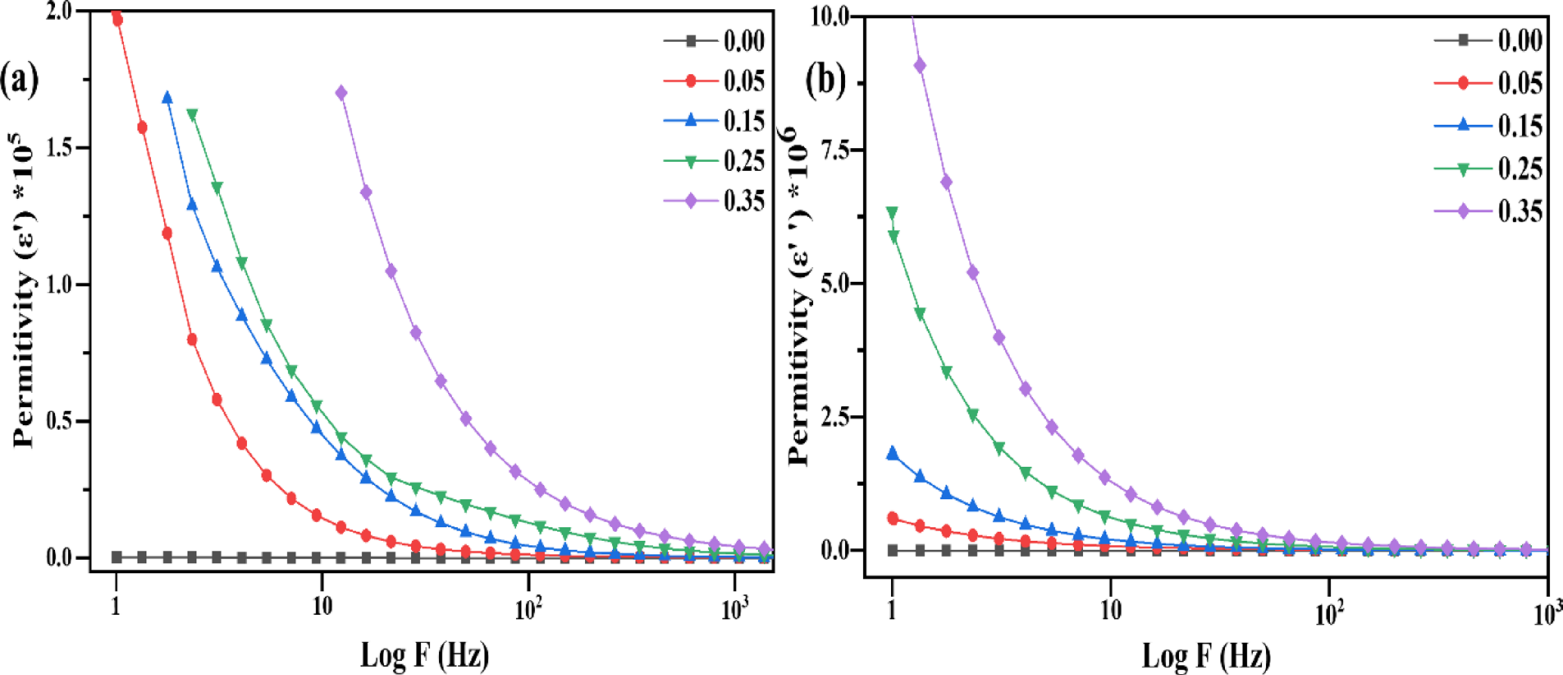
**(a)** Real part of the dielectric constant (ε’), **(b)** Imaginary part of the dielectric constant (ε’’).

In the dielectric study, the permittivity (real and imaginary) of Sr_x_Zn_1−x_Fe_2_O_4_, prepared by the co-precipitation method, is revealed in Fig. 10, which was examined at room temperature. The real part of Fig. 10**(a)** measures the dipole alignment strength and energy stored in the dielectric medium, while the imaginary part corresponds to energy dissipation (loss factor). Together, they represent the total polarization of the dielectric materials. Figure 10**(b)** illustrates the imaginary component, highlighting energy dissipation.

Impedance plots demonstrate a clear trend with increasing Sr²⁺ doping. Higher doping concentrations improve dielectric performance, as reflected in the semicircular curves. These curves suggest reduced resistance and uniform particle size; distinguishing the contributions from grain boundaries and grains is challenging. Variations in local structural features, such as phase transitions and defects, can lead to multiple dipolar relaxation dynamics, complicating the separation of phases in impedance analysis (ε´´ vs. ε´).

For all samples, dielectric curves exhibit strong dispersion and asymmetry at lower frequencies, characteristic of non-Debye dielectric behavior^20^. As the doping concentration increases, the arc angle with the abscissa increases, indicating enhanced material polarization. Additionally, low-frequency dispersion is observed in all samples, with the dispersion region expanding at higher frequencies as the doping concentration increases, signifying an enhanced dielectric response. In Fig. 10**(b)**, the imaginary permittivity shows exponential decay with increasing frequency for all samples, indicating a significant relaxation process. The sample with the highest doping concentration (x = 0.35) shows higher permittivity at lower frequencies, indicating enhanced polarization, which gradually weakens with increasing frequency. The dielectric permittivity (′ and ε′′) is displayed up to 10^3^ Hz due to technical restrictions that prevented reliable readings beyond this frequency; also, the data curve moves parallel to the abscissa. In contrast, modulus, AC conductivity, and loss tangent were all consistently measured up to 10^7^ Hz. This study primarily examines frequency-dependent dielectric behavior at room temperature. The absence of studies on thermal stability and reproducibility in this work is a shortcoming and a crucial area for further investigation. The practical integration of spinel ferrites into electronic and energy storage systems depends on their stability throughout different heat cycles and their repeatability under repeated measurements. Sr-substituted ZnFeO₄ exhibits a structurally driven dielectric boost in addition to compositional enhancement. The observed decrease in dielectric loss and enhancement of ε′ are directly related to defect-assisted polarization and cation redistribution caused by Sr. While XRD analysis shows lattice compression and micro-strain creation with increased Sr content, Mössbauer spectroscopy supports Fe³⁺ migration toward B-sites, amplifying local electric dipole moments. In contrast to the typical dipolar response in undoped ferrites, the strain-assisted Maxwell-Wagner relaxation mechanism is introduced by the interaction between structural distortion and charge carrier hopping. The optimized Sr₀.₃₅Zn₀.₆₅Fe₂O₄ composition exhibits optimal permittivity and low loss up to 10⁷ Hz, making it a promising option for energy storage systems and high-frequency dielectric devices.

### Dielectric modulus of SZF composites

**Fig. 11:**
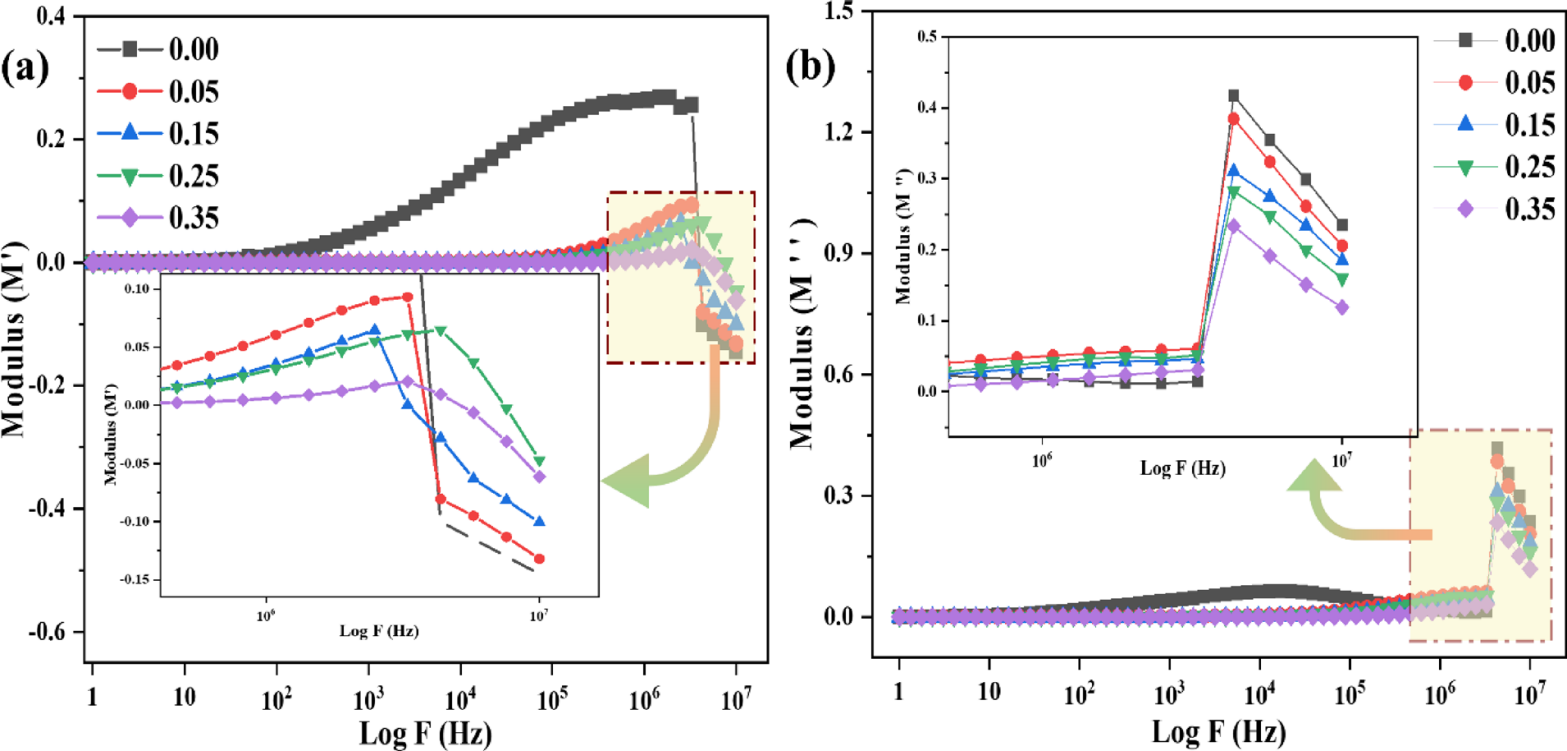
(**a**) Real part of the dielectric modulus (M ‘), (**b**) Imaginary part of the dielectric modulus (M ‘’).

The M′ spectra of the Sr_x_Zn_1−x_Fe_2_O_4_ samples in the present investigation exhibit minimal values at lower frequencies for all doping levels (x = 0.00 - 0.35), pointing to the dominance of long-range conduction and the absence of restorative force, as shown in Fig. 11**(a)**. The initiation of short-range relaxation processes and a confirmation of a change in the response from interfacial to bulk polarization are indicated by the rise in M′ with increasing frequency. This onset moves to higher frequencies as Sr^2+^ doping increases, suggesting that grain interiors are becoming more resistive and that dielectric shielding is decreasing. Following a non-Debye relaxation, the modulus spectra observed a sigmoidal shape, indicating a spread in relaxation times that is probably caused by compositional inhomogeneities, defect clusters, and lattice distortion^25^.

Whereas, for determining the relaxation dynamics in the system, the imaginary component of the electrical modulus (M″) is crucial, particularly in determining the position and shape of peaks that represent the time constants of dipolar motion or localized hopping, as shown in Fig. 11**(b)**. As the Sr content increases, the M″ spectra of all samples exhibit distinct relaxation peaks that shift to higher frequencies, indicating a decrease in relaxation time (τ) and a transition toward faster charge carrier dynamics. According to the CBH (correlated barrier hopping) model, this behavior supports hopping conduction by implying that Sr²⁺ substitution produces defect states or modifies Fe³⁺ ↔ Fe²⁺ exchange channels. The non-Debye type relaxation, which is defined by a distribution of relaxation times, is supported by the asymmetric and widened peaks. These results are in good agreement with the dielectric and impedance data, confirming the notion that increased Sr doping alters the charge distribution and enhances dielectric relaxation^25,26^. The M′ spectra show somewhat negative values in the high-frequency band (see inset, Fig. 11a). These numbers do not represent inherent material behavior; rather, they are the result of instrumental artifacts at the measurement border, such as stray capacitances and phase correction errors. Moreover, extrinsic effects take over after the abrupt transitions seen at ~ 10⁶ - 10⁷ Hz, which indicate the limit of bulk dielectric relaxation. Therefore, only the reliable mid-frequency data are discussed, and these regions were not included in the quantitative relaxation study^27,50,51^.

### Electrical conductivity of SZF composites

**Fig. 12:**
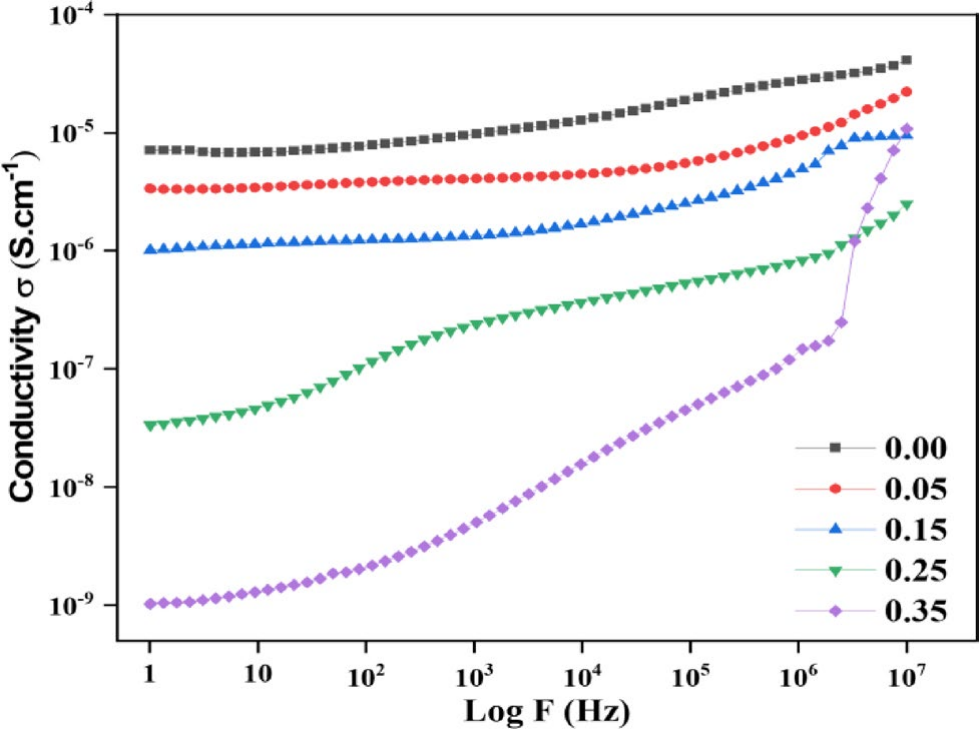
Conductivity of strontium-doped Zn-Fe nano composite.

The frequency-dependent AC conductivity (σ) of SrₓZn₁₋ₓFe₂O₄ ferrites with different Sr²⁺ contents (x = 0 - 0.35) is shown in Fig. 12, and it satisfies the requirements for good dielectric behavior. Conductivity is shown to decrease systematically over the frequency range, notably in the low-frequency region, as Sr doping increases, especially above x = 0.15. This is a crucial criterion for effective dielectric materials. At low frequencies, the conductivity of the sample with x = 0.35 is the lowest, extending down to the 10⁻⁹ S·cm⁻¹ range. This suggests that the sample has excellent insulating properties and little leakage current. Such behavior indicates improved grain boundary resistance and decreased charge carrier mobility, both of which inhibit dielectric loss. Additionally, under non-Debye-type relaxation, the frequency-dependent dispersion points to hopping conduction mechanisms connected to localized charge carriers. This conductivity profile demonstrates that increased Sr^2+^ doping enhances polarization stability and reduces energy dissipation, which improves dielectric performance^28^. Figure 12 displays the frequency-dependent AC conductivity (σ) of SrₓZn₁₋ₓFe₂O₄ (x = 0 - 0.35). Improved insulating behavior is indicated by a systematic decrease in conductivity with increasing Sr2⁺ substitution, particularly beyond x = 0.15. For x = 0.35, the lowest conductivity, roughly 10⁻⁹ S·cm⁻¹ at low frequencies, indicates poor charge carrier mobility and significant grain boundary resistance. The dielectric’s performance is improved by this decrease in leakage current. A non-Debye-type relaxation mechanism controlled by polaronic hopping between localized Fe²⁺/Fe³⁺ ions is confirmed by the observed dispersion in σ(ω) with frequency. Therefore, Sr doping stabilizes polarization and decreases energy dissipation by changing the Fe-O-Fe super-exchange pathways and raising potential barriers at grain boundaries, which effectively regulates charge transport.

### Tan loss

**Fig. 13:**
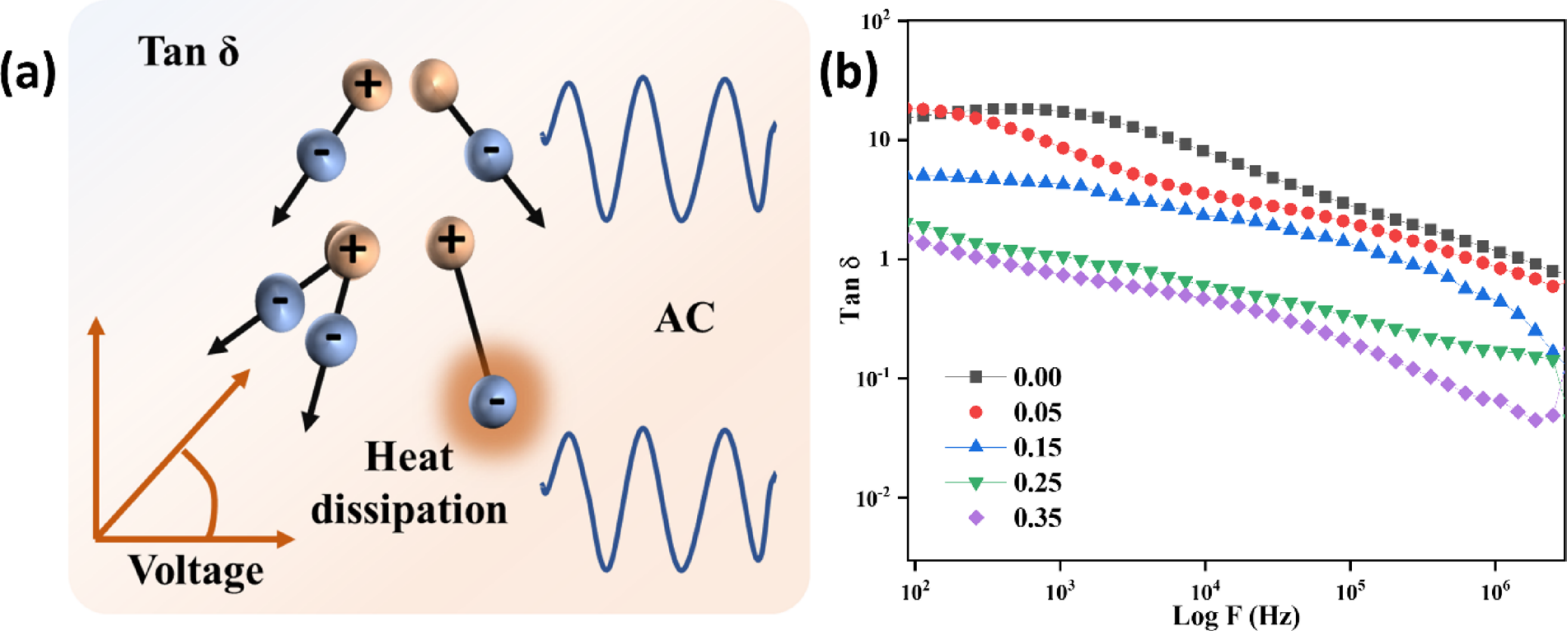
Delay in dipole orientation and heat dissipation studied by Tan Loss of SZF nano composite (**a**) Schematic representation of tan loss and (**b**) observation of tan loss by experimentally determined data.

The dielectric loss (tan δ) in a ferrite material under an AC electric field is seen in Fig. 13**(a)**, schematic. Numerous dipoles react to the oscillating field with a delay caused by polarization lag, lattice imperfections, and interfacial phenomena, leading to energy dissipation. Due to inherent variables including lattice defects, space charge effects, grain boundary impedance, and interfacial polarization (Maxwell-Wagner type), these dipoles try to follow the AC field’s quickly changing direction, but their orientation is delayed. It is important to note that the vector diagram does not represent Ohmic conduction; rather, it represents capacitive reactance with loss. Dielectric energy dissipation resulting from delayed dipolar alignment under alternating fields is quantified by the tan δ value. The analyzed variation in dielectric loss (tan δ) with frequency for the Sr_x_Zn_1−x_Fe_2_O_4_ system, as shown in Fig. 13**(b)**, synthesized via co-precipitation, exhibits a pronounced frequency-dependent relaxation behavior consistent with interfacial polarization governed by the Maxwell-Wagner and Koop’s phenomenological models^28^. At lower frequencies, elevated tan δ values for the undoped and lightly doped samples (x = 0, 0.05) indicate significant space charge accumulation at grain boundaries, attributed to interfacial charge carrier hopping and defect-induced relaxation, as mentioned earlier^18^. As the frequency increases, the observed sharp decrease in tan δ arises from the inability of charge carriers and dipoles to align synchronously with the rapidly alternating field, leading to reduced dielectric losses. Among all samples, the x = 0.35 composition exhibits the lowest tan δ values across the entire frequency range, denoting exceptional dielectric quality, minimal energy dissipation, and optimal charge confinement. The overall decreasing trend in tan δ with increasing Sr²⁺ content correlates with improved microstructural homogeneity, reduced oxygen vacancy concentration, and effective suppression of grain boundary leakage, establishing x = 0.35 as the most dielectric-efficient composition in the present series.

### Comparison of this study

Strontium-doped zinc ferrite nanoparticles (Sr_x_Zn_1−x_Fe_2_O_4_) prepared by the co-precipitation method at a constant annealing temperature exhibit outstanding performance compared to prior data, as indicated in Table 4. This illustrates the efficacy of this strategy, with well-controlled crystal size, adjusted lattice parameters, and improved semiconductor behavior.

**Table 4:** The properties of the synthesized (Sr_x_Zn_1-x_Fe_2_O_4_) sample are compared with other recently published data.

S.no.	Material	Temp. (°C)	Annealing Time (min)	Crystal size (nm)	Dielectric	Tan loss	Band Gap	Ref.
1.	Co_x_Zn_1−x_Fe_2_O_4_ x = 0.9	800	300	58	-	-	2.3	^21^
2.	ZnFe_2_O_4_	600	360	51	-	10^4^ − 10^3^	-	^25^
3.	Ni_1 − x_Zn_x_Fe_2_O_4_ x = 0.5	1200	720	72	10^5^ − 10^3^	10^4^ − 10^3^	2.83	^27^
4.	Ni_x_Zn_1−x_Fe_2_O_4_ x = 0.8	500	240	34	10^5^ − 10^3^	10^4^ − 10^2^	-	^30^
5.	SrZnFe_2_O_4_	550	300	39	10^5^ − 10^3^	10^4^ − 10^3^	2.5	^31^
6.	PdOSrFe_2_O_4_	650	120	17	10^4^ − 10^2^	10^5^ − 10^3^	2.8	^32^
7.	NiSrFe_2_O_4_	600	360	45	10^4^ − 10^2^	10^4^ − 10^2^	2.9	^34^
8.	SrZnFe_2_O_4_	700	480	38	10^5^ − 10^3^	10^5^ − 10^3^	2.0	^22^
9.	In_0.3_Zn_0.8_Fe_2_O_4_	700	480	21	10^5^ − 10^3^	10^5^ − 10^3^	2.0	^50^
10.	Sr_x_Zn_1−x_Fe_2_O_4_ x = 0.35	800	480	58	10^5^ − 10^3^	10^6^ − 10^2^	2.2	This work

## Conclusion

In a nutshell, Strontium substitution in ZnFe₂O₄ markedly affects the material’s structural, optical, magnetic, and dielectric properties. The development of a single-phase cubic spinel structure with robust metal–oxygen bonding is confirmed by XRD, FTIR, and Raman studies. According to UV-Vis spectra, the material’s optical band gap progressively drops from 2.8 eV (x = 0) to 2.2 eV (x = 0.35), putting it in the ideal semiconducting range for optoelectronic devices. Site-specific Fe³⁺ occupancy is shown by Mössbauer spectroscopy, and cation redistribution is confirmed by the relative B-site percentage dropping by about 15% as Sr concentration rises. Permittivity (ε′) reaches a maximum of almost 185 at low frequencies with x = 0.35, according to dielectric studies, whereas the dielectric loss tangent falls to 0.05 at high frequencies (> 10⁶ Hz), indicating stability and minimal energy dissipation. When Sr is added, the AC conductivity drops by almost an order of magnitude (from 10⁻⁵ to 10⁻⁶ S/cm), indicating improved dielectric integrity. All of these findings make SrₓZn₁₋ₓFe₂O₄ a promising option for dielectric applications such as capacitors, high-frequency transformers, and sensing devices, as well as optoelectronic devices functioning in the visible spectrum.

## Data Availability

All the data generated during the present investigation are presented within the manuscript.
